# How emotional expression in human-like virtual influencers drives user engagement: empathy model and its antecedents

**DOI:** 10.3389/fpsyg.2025.1544037

**Published:** 2026-01-06

**Authors:** Rongqin Liu, Xiaofen Li, Shaojian Liu

**Affiliations:** 1 College of Business, Nanning University, Nanning, China; 2 China Energy Engineering Group Guangxi Electric Power Design Institute Co., Ltd., Nanning, China

**Keywords:** human-like virtual influencers, emotional expression, user engagement, users’ perceptions, empathy model

## Abstract

**Introduction:**

This study builds upon the rapid advancement of artificial intelligence and influencer marketing to examine the impact of emotional expressions by human-like virtual influencers on user engagement. Utilizing the empathy model and its antecedents, it investigates how users’ perceptions—specifically perceived attractiveness, authenticity, escapism, and presence—affect both cognitive and affective empathy, which, in turn, influence user engagement.

**Methods:**

A within-subjects experimental design was employed to test the proposed model. The study involved 118 participants who were exposed to six distinct emotional stimuli exhibited by virtual influencers.

**Results:**

The findings reveal significant linear effects of perceived authenticity and presence on both cognitive and affective empathy. Moreover, perceived attractiveness and escapism were found to jointly enhance users’ cognitive and affective empathy. The study also uncovers mediating effects of cognitive and affective empathy on user engagement. Subgroup analysis highlights the considerable impact of human-like virtual influencers’ emotional expressions, particularly positive emotions, on user perceptions and empathy. Conversely, extreme negative emotions, such as anger, were found to reduce empathy through the perception of escapism.

**Discussion:**

These results contribute to a deeper understanding of the mechanisms driving user engagement with human-like virtual influencers and suggest strategies for enhancing engagement through emotional expression. The study provides valuable insights for the design and implementation of virtual influencers in marketing strategies, emphasizing the critical role of emotional expression in fostering meaningful user connections.

## 1 Introduction

The burgeoning realm of social media has catalyzed the ascendancy of influencer marketing, a strategy that harnesses the clout of individuals with substantial followings, often termed opinion leaders or internet celebrities, on various platforms to endorse products or services (Melnychuk et al., 2024; Venciute et al., 2023). These influencers command large, trusting audiences in specific domains, significantly swaying their followers’ perceptions and purchasing behaviors (Belanche et al., 2024; Bu et al., 2022). As social media continues to dominate communication channels, brands have increasingly recognized the strategic importance of influencer partnerships, positioning influencer marketing as a critical element of their development strategies (Allal-Chérif et al., 2024; Gutuleac et al., 2024). According to a report by Statista, a global statistical database, the global market for influencer advertising will burgeon to $52 billion by 2028 (Statista, 2023).

Parallelly, advancements in artificial intelligence and virtual reality have given rise to human-like virtual influencers, a novel cadre of social media personalities (Park G. et al., 2021; Kim and Park, 2024). Created through sophisticated computer-generated imagery, these virtual entities boast highly anthropomorphized appearances and engage actively on social platforms, sharing glimpses into their fabricated lives and vocations (Kim and Park, 2023). Due to their low production costs and absence of risks related to scandals, virtual influencers offer an attractive option for brands seeking commercial partnerships (Franke et al., 2023; Kim et al., 2023). From online customer service and educational assistance to entertainment and medical consultations, the applications of virtual influencers are expanding rapidly. Despite significant technological advancements, however, the quality of interaction between virtual influencers and human users continues to be a critical determinant of user experience (Ham et al., 2023; Moustakas et al., 2020). Notably, the inherently “authentically fake” quality of virtual influencers has prompted their management teams to emphasize emotional expressions on social media (Lou et al., 2023; Park G. et al., 2021). Emotional expression is a critical facet of human-machine interaction and a fundamental connector for enhancing user engagement (Esposito et al., 2015; Li et al., 2024). Therefore, the emotional expression of virtual influencers plays an indispensable role in enhancing the authenticity and immersion of such interactions (De Brito Silva et al., 2022; Li et al., 2024). However, since human-like virtual influencers typically engage with users through pre-programmed scripts, their real-time, dynamic interactions are often less responsive compared to those of real influencers (Wan and Jiang, 2023). Despite this limitation, users remain willing to engage with these virtual influencers on social media. This underscores the importance of examining how the emotional expressions of human-like virtual influencers impact user engagement.

Further, past research indicates that the acceptance of virtual influencers by users largely depends on the users’ empathy, which involves the ability to understand and share the experiences, emotions, and thoughts of others (Allal-Chérif et al., 2024; Mirowska and Arsenyan, 2023). Researchers categorize empathy into cognitive and affective components: cognitive empathy involves understanding others’ thoughts and feelings and taking appropriate actions, whereas affective empathy entails the capacity to feel what another person is experiencing emotionally (Barnett and Mann, 2013; Gladstein, 1983; Ventura et al., 2020). When virtual influencers express emotions on social media, cognitive empathy helps users to comprehend these emotions and intentions more deeply, enhancing their emotional connection with the content and leading to positive behavioral feedback such as purchase intentions (Ioannidou and Konstantikaki, 2008; Malär et al., 2011). The power of affective empathy, however, lies in its ability to strengthen the emotional bond between users and virtual entities, thereby deepening their engagement with the virtual content and encouraging active interaction (Paiva et al., 2017; Shin, 2018). When users resonate emotionally with virtual influencers, this connection not only enhances their bond with the content but also motivates further engagement and interaction (Mirowska and Arsenyan, 2023).

Additionally, drawing on empathy-related literature, prior studies have shown that user perceptions (e.g., perceived attractiveness) can influence users’ empathic responses (Fisher and Ma, 2014; Zhu et al., 2022). Hence, this study aims to explore the intricate relationship between the emotional expressions of virtual influencers and the resulting user perceptions, empathy and engagement. Specifically, we focus on four perceptual dimensions (i.e., perceived attractiveness, authenticity, escapism, and presence) that have been frequently highlighted in prior literature on human-computer interaction and virtual characters (Kim and Baek, 2024; Kim and Park, 2023; Kim and Park, 2024; Mirowska and Arsenyan, 2023). These dimensions were selected based on their demonstrated relevance in shaping users’ emotional responses and immersive experiences in human-like virtual environments. Hence, this study aims to explore how emotional expressions by human-like virtual influencers shape users’ perceptions, and how these perceptions influence cognitive and affective empathy, which ultimately drive user engagement. The specific research questions are as follows:

*RQ1*: How does the human-like virtual influencers’ emotional expression impact users’ perceptions?

*RQ2*: How do the users’ perceptions affect user engagement through cognitive and affective empathy?

By doing so, our work makes several key contributes to the literature in the following ways. First, it expands influencer marketing literature by focusing on the emerging domain of human-like virtual influencers. Second, it complements existing research on emotional expression by investigating how it shapes user perceptions in non-human contexts. Third, it enriches the empathy literature by analyzing how specific user perceptions act as antecedents to empathy, and how empathy mediates the link between emotional expression and engagement, offering both theoretical insight and practical guidance for brands deploying virtual influencers.

## 2 Related work

### 2.1 Emotional expression of influencers and user engagement

Prior research indicates that emotional expression serves as a critical content cue enabling social media influencers (SMIs) to shape the perceptions and behaviors of their audience (Kay et al., 2023). Initially, studies predominantly concentrated on fundamental aspects of emotional expression, delineating distinctions between positive and negative emotions (Chun et al., 2021; Gerbaudo et al., 2019; Ibrahim et al., 2017). For example, Weismueller et al. (2022) demonstrate that SMIs’ positive emotional displays can provoke favorable user responses, such as likes, comments, and shares. Conversely, while negative emotions also garner attention, they typically result in reduced user engagement (Khobzi et al., 2019). These investigations lay the groundwork for a nuanced understanding of how emotional expression influences user engagement.

As scholarly inquiry advances, researchers are examining more complex aspects of emotional expression, including emotional intensity and complexity (Cascio Rizzo et al., 2024; Rietveld et al., 2020). They propose that emotional expression transcends simple categorization into positive or negative, encompassing both the intensity and diversity of emotions. Studies reveal that high emotional intensity significantly boosts user engagement, whereas emotional diversity appeals to a broader audience. Moreover, certain studies have assessed the variable impacts of specific emotional expressions across different social media platforms (Aldous et al., 2023; Park et al., 2023; Waterloo et al., 2018). Beyond merely analyzing the content of emotional expression, researchers are delving into underlying factors that significantly influence user engagement in online settings (Lechner and Paul, 2019; Zloteanu and Krumhuber, 2021). For instance, Zloteanu and Krumhuber (2021) suggests that users are more likely to interact with others when they perceive the emotional expressions as authentic and trustworthy. This authenticity is reflected not only in the consistency and coherence of the expressions but also in the interaction dynamics between SMIs and their followers (Gross et al., 2023; Ren et al., 2023; Sánchez-Fernández and Jiménez-Castillo, 2021). Additionally, Chen et al. (2025) has shown that well-established quasi-social relationships enhance users’ receptiveness to emotional expressions. These studies indicate that there are a series of factors that can affect the relationship between influencers’ emotional expression and user engagement.

### 2.2 Human-like virtual influencers

In the digital era, virtual influencers have emerged as a pivotal component of social media marketing (Park et al., 2024). These digital personas are distinctly categorized into cartoon-like (e.g., Luo Tianyi), animal-like (e.g., Mao Baozai), and human-like virtual influencers (e.g., Lil Miquela), catering to diverse audience preferences (Arsenyan and Mirowska, 2021). Among these categories, human-like virtual influencers have garnered significant attention (Kim et al., 2023; Qu and Baek, 2024; Zhou et al., 2024). Their appeal lies in their anthropomorphic features and realistic aesthetics, which not only foster a sense of familiarity but also enable deeper emotional connections with their audiences (Shao, 2024; Wan et al., 2024). This connection is critical in establishing trust and emotional bonds, essential elements for effective marketing and brand engagement (Jiang et al., 2024; Sands et al., 2022).

Extensive research has been conducted to understand the dynamics between human-like virtual influencers and consumer behavior. One primary attribute is perceived attractiveness, which plays a significant role in retaining audience attention (Chaihanchanchai et al., 2024; Park et al., 2023). Attractiveness encompasses a broad spectrum including personality traits, voice quality, and interactive capabilities (Kim and Park, 2023). Human-like virtual influencers that embody a charismatic and appealing persona are more likely to captivate their audience, leading to more successful endorsements and sustained brand partnerships. Authenticity is another critical factor in the success of human-like virtual influencers (Lee et al., 2025; Li and Ma, 2024). When these digital entities exhibit genuine characteristics and behaviors, they resonate more profoundly with users (Allal-Chérif et al., 2024; De Brito Silva et al., 2022). Authenticity manifests through consistent and relatable communication, the sharing of genuine content, and maintaining a persona that reflects true human interactions (Kim and Baek, 2024). This authenticity fosters trust and can significantly enhance user engagement and brand loyalty (Koles et al., 2024; Lou et al., 2023). In addition to attractiveness and authenticity, the immersive experiences provided by human-like virtual influencers play a crucial role in modern marketing. These influencers offer an escape from the pressures of daily life, providing psychological comfort and entertainment (Mirowska and Arsenyan, 2023). In today’s fast-paced world, where individuals often seek brief moments of relaxation and enjoyment (Freund, 2025), engaging and entertaining content becomes a source of comfort and joy for followers. The concept of perceived presence is pivotal in strengthening the human-computer interaction (Kim and Park, 2024; Yan et al., 2024). The ability of human-like virtual influencers to respond promptly and engage in personalized interactions significantly enhances the overall user experience (Stein et al., 2024). Real-time responses and tailored interactions make users feel valued and heard, crucial for building long-term relationships and fostering a loyal fan base (Ahn et al., 2022; Wang et al., 2024). Beyond these perceptions, empathy also plays a transformative role in deepening the connections between human-like virtual influencers and their audience (Jin, 2023; Mirowska and Arsenyan, 2023). Empathy involves the ability to understand and share the feelings of another, creating a strong emotional connection. When human-like virtual influencers successfully evoke empathy, they foster a more profound emotional bond with users (Bouchard et al., 2013; Davlembayeva et al., 2024). This connection is facilitated by sharing relatable content, engaging with users in a thoughtful manner, and aligning the influencer’s narratives with the values and emotions of the audience.

In addition, to our best knowledge, only three studies have explored the impact of human-like virtual influencers’ emotional expression on user engagement. Specifically, Li et al. (2024) investigated whether users’ attitudes can be transformed into behavior based on emotional expression and found that the emotional expressions impacting user behavior range from lust, happiness, sadness, to no emotion. Ham et al. (2024) suggested that human-like virtual influencers should avoid displaying negative emotions, which can lead to low perceived emotional intelligence, authenticity, and user attitude. Yu et al. (2024) confirmed that emotional expression plays an important role in triggering user engagement, focusing on images shared by human-like virtual influencers on social media without exploring textual content. Therefore, current research on how emotional expression of human-like virtual influencers affects user engagement is still in its early stages.

### 2.3 Empathy model

Empathy, as a core mechanism in interpersonal interactions, has been systematically studied in the field of psychology for nearly half a century. It is defined as the ability of an individual to recognize, understand, and share the experiences, emotions, and feelings of others (Cuff et al., 2016; Paiva et al., 2017). According to the multidimensional model proposed by Davis (1983), empathy consists of two basic dimensions: cognitive empathy and affective empathy. This framework has been widely validated and expanded in subsequent studies (Gladstein, 1983; Barnett and Mann, 2013). Neuroscientific research has further revealed the existence of a dual-channel mechanism: cognitive empathy primarily activates the prefrontal cortex and involves inferring others’ mental states and adopting their perspectives (Decety and Jackson, 2004); while affective empathy is associated with the mirror neuron system, manifesting as automatic resonance with the emotional states of others (Singer and Lamm, 2009).

In the context of modern social media and the influence economy, empathy serves as the key link in building and strengthening the close connection between influencers and users (Jung and Im, 2021). From the user’s perspective, through cognitive empathy, they are able to gain insight into the narrative logic and emotional appeals behind the influencer, which is a crucial step in deepening the understanding of the influencer (Dovzhik et al., 2021). Research has shown that users’ personal traits (e.g., openness, emotional stability), prior experiences, cultural backgrounds, social environments, and technology usage habits significantly influence their empathetic responses to influencers (Li et al., 2023; Zhou et al., 2021). In addition, other psychological reactions, such as users’ cognitive biases, emotional states, self-identity, and social comparison tendencies, also play a crucial role in shaping their empathy (Reed II and Forehand, 2016). For example, users’ emotional states (e.g., anxiety and joy) may either amplify or suppress their empathetic responses (Beale and Creed, 2009). Furthermore, whether users identify with the influencers’ values or lifestyle can strengthen or weaken their empathy (Brewster and Sklar, 2022). At the same time, social comparison processes (e.g., self-worth, admiration, and envy) between users and influencers can also impact the intensity of emotional resonance (Jin and Ryu, 2020).

When influencers express emotions on social media, users, by decoding these emotional signals and their contextual meanings, are able to construct a cognitive model of the influencer’s emotional state (Holiday et al., 2023). If this expression is genuine and compelling, it enhances the user’s sense of social identification and perception of the influencers’ attractiveness, further stimulating the user’s cognitive empathy (Kay et al., 2023). At the same time, if the influencer’s emotional expression resonates with the user’s inner world and meets their specific psychological needs—such as relieving stress through humor, evoking sympathy through sadness, or inspiring determination through motivational stories (Liu-Thompkins et al., 2022)—it can trigger the user’s affective empathy, causing them to resonate with the influencer or content on an emotional level. This reduces social isolation and enhances the user’s participation and engagement.

In the emerging field of virtual influencers, empathy also plays an indispensable role in understanding and explaining the interactive relationship between virtual influencers and users (Mirowska and Arsenyan, 2023; Zhang et al., 2025). When users successfully understand the emotional information conveyed by virtual influencers through cognitive empathy and establish an emotional connection with them through affective empathy, they are more likely to actively engage in social media activities, such as liking, commenting, sharing content, and even engaging in deeper interactions. It is this power of emotional resonance that greatly motivates user participation, prompting them to form close interactions with content based on emotional synchronization, thus constructing a rich and diverse emotional interaction network in the intersecting social spaces of the virtual and the real. Although previous studies have revealed the important role of empathy in the relationship between virtual influencers and users, research on which psychological factors influence the relationship between virtual influencers and user participation through the empathy model remains insufficient.

### 2.4 The contribution of this research

This paper investigates the interplay between user perception (i.e., perceived attractiveness, authenticity, escapism, and presence), cognitive empathy, and affective empathy, alongside the resultant effects on user participation, thereby elucidating the mechanisms through which the emotional expression of human-like virtual influencers changes user engagement. The research commences with an examination of the linear impacts of perceived attractiveness, authenticity, escapism, and presence on cognitive empathy using linear regression analysis. Following this, the study delves into the aggregated influence of these user perception interactions on cognitive empathy. Furthermore, the variance in the effects of these user perceptions on cognitive empathy across different emotional expression is scrutinized through subgroup regression analysis. This methodological framework is similarly applied to assess the relationship between user perception and affective empathy. Finally, we expanded our exploration scope to understand how cognitive empathy and affective empathy affect user engagement, thereby providing a comprehensive analysis of the pathways through which these perception dimensions interact with user participation.

## 3 Hypothesis development

**Figure 1:**
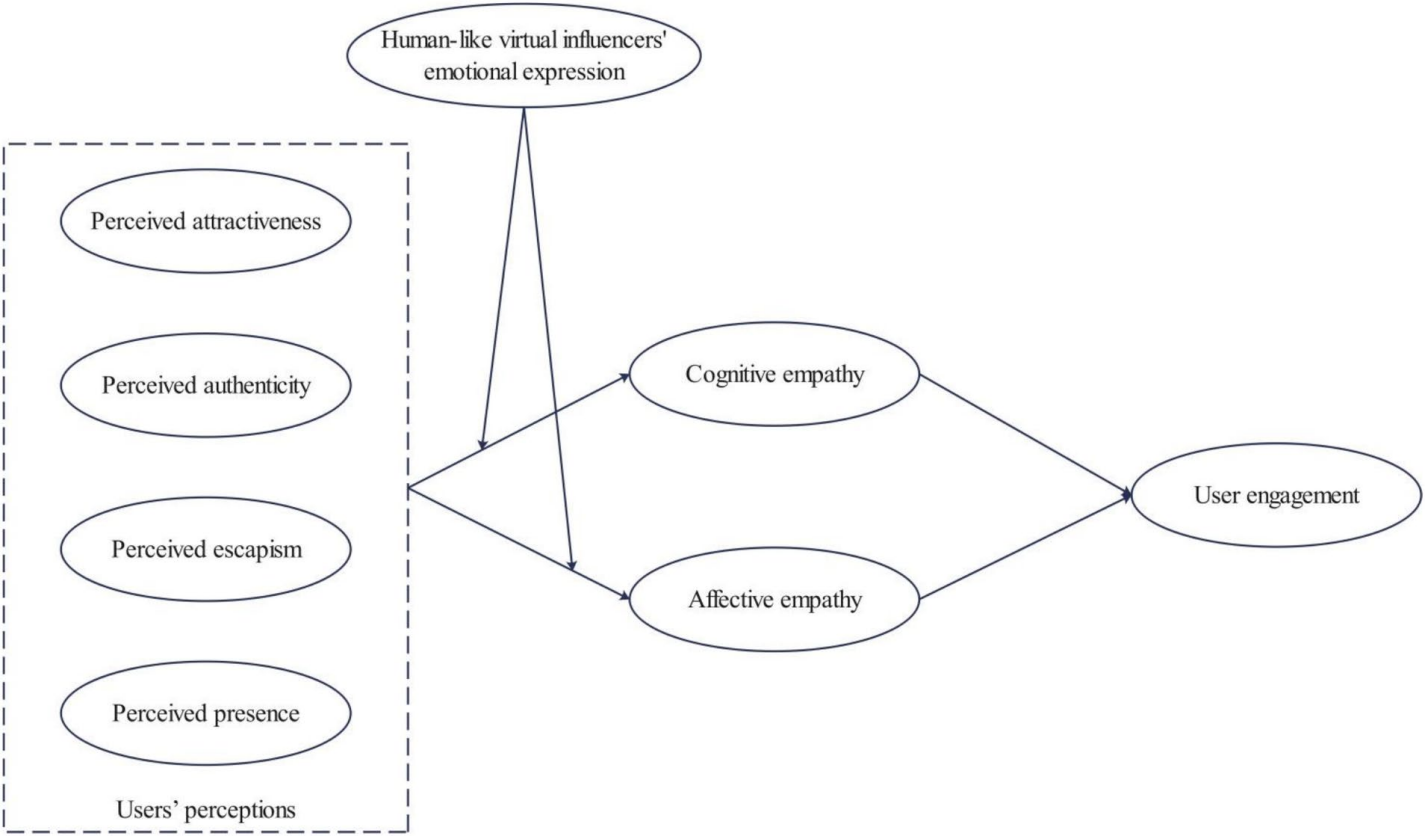
The research model.

Our research aims to elucidate how the emotional expression of human-like virtual influencers impact user engagement through user perception (i.e., perceived attractiveness, authenticity, escapism, and presence) and empathy model (i.e., cognitive empathy and affective empathy). The research model was depicted in Figure 1.

### 3.1 User perception and cognitive empathy

#### 3.1.1 Perceived attractiveness and cognitive empathy

Perceived attractiveness plays a crucial role in shaping users’ preferences toward content delivered by human-like virtual influencers (Kim and Park, 2023). It encompasses various factors, including the visual appeal of the influencer’s appearance, their personality traits, content quality, and emotional expressions. The creation of this attractiveness is influenced by a combination of elements, such as the novelty of the content, the authenticity of emotional expressions, and the alignment with users’ personalized needs and interests. Previous research suggests that human-like virtual influencers with high perceived attractiveness (e.g., those featuring anthropomorphic traits or natural emotional expressions) are more likely to capture users’ visual attention (Kuo and Le, 2025). This increased attention prompts users to actively decode emotional cues—such as micro-expressions, tone of voice, and non-verbal behaviors—thereby engaging in psychological simulation through social cognition templates. Such interactions enhance users’ understanding and recognition of emotional signals. Furthermore, perceived attractiveness enhances the credibility of the virtual influencer, stimulating users’ motivations for para-social interaction (Park and Sung, 2023). This, in turn, lowers the cognitive threshold for interpreting the emotional intentions of the virtual influencers. This process of “rational empathy” relies on cognitive reasoning, driven by attractiveness, ultimately facilitating users’ accurate understanding of the virtual influencers’ emotional state. Based on this rationale, the study posits the following hypothesis:

*H1*: Perceived attractiveness is positively related to cognitive empathy.

#### 3.1.2 Perceived authenticity and cognitive empathy

Perceived authenticity refers to users’ cognitive assessment of the authenticity, credibility, and sincerity of a human-like virtual influencers’ emotional expressions (Lou et al., 2023). When users perceive the emotional expressions of a virtual influencers as authentic, they are more likely to engage their social cognition mechanisms, such as logical reasoning and perspective-taking, thereby enhancing cognitive empathy. Authentic emotional cues, such as contextually appropriate microexpressions and tonal variations, provide users with clear emotional logic, encouraging them to infer the virtual influencers intentions or needs through the “theory of mind” framework (Cornelius et al., 2023; Kim and Baek, 2024). This perception of authenticity reduces the cognitive uncertainty users experience in virtual interactions, making them more inclined to invest cognitive resources in understanding the causal relationships underlying the emotional expressions (Ozdemir et al., 2023; Yu et al., 2024). The depth of cognitive empathy, therefore, largely depends on the emotional interpretability afforded by perceived authenticity, which entails users’ rational agreement with the question of “why the emotional expression is reasonable.” Based on this, the following hypothesis is proposed:

*H2*: Perceived authenticity is positively related to cognitive empathy.

#### 3.1.3 Perceived escapism and cognitive empathy

Perceived escapism refers to the psychological state in which users, through their interactions with human-like virtual influencers, seek temporary relief from the pressures of everyday life (Verhagen et al., 2012). This desire for escape motivates users to engage with virtual content that offers psychological comfort and emotional support, allowing them to experience emotional release and mental calmness (Ekman and Halpern, 2015). Perceived escapism, facilitated through mechanisms of situational immersion and psychological detachment, indirectly enhances users’ cognitive empathy toward virtual influencers. When users temporarily escape real-world stress through the emotional expressions of virtual influencers, their attention becomes highly focused on the logical construction of the virtual scenario (Mirowska and Arsenyan, 2023). This heightened focus prompts users to actively interpret the emotional motivations of the virtual influencers. Such psychological detachment from reality reduces users’ tendency to critically assess complex real-world issues, making them more receptive to the internal logic of the virtual narrative. Through cognitive empathy, users actively engage with the emotional logic of the story (Kang et al., 2020). This escapism-driven engagement functions as an implicit form of training, enhancing users’ real-world cognitive abilities through virtual empathy. Thus, we propose the following hypothesis:

*H3*: Perceived escapism is positively related to cognitive empathy.

#### 3.1.4 Perceived presence and cognitive empathy

Perceived presence refers to the psychological state in which individuals feel socially accepted and valued, experiencing a sense of belonging to a community (Kim and Park, 2024). In virtual environments, presence significantly influences how users perceive their role and value within the virtual community (Tsai and Bagozzi, 2014). When interacting with virtual influencers, presence denotes the experience where users feel that their participation is meaningful and significant. This sense of presence emerges from interactions with virtual influencers or other community members, enhancing social belonging, self-worth, and social identity (Ahn et al., 2022). When users perceive virtual influencers as real entities with autonomous emotional expressions, they engage in mentalization, analyzing the underlying logic of these emotional cues. Presence also reduces cognitive dissonance, making users more likely to adopt an observer’s perspective and reason through the appropriateness of emotional expressions (Jeong et al., 2019). Based on this, we propose the following hypothesis:

*H4*: Perceived presence is positively related to cognitive empathy.

### 3.2 User perception and affective empathy

#### 3.2.1 Perceived attractiveness and affective empathy

Perceived attractiveness plays a critical role in triggering affective empathy toward virtual influencers by directly influencing emotional contagion and physiological arousal. Highly attractive virtual influencers, such as those with vivid facial expressions or aesthetically pleasing features, activate the user’s mirror neuron system, leading to unconscious mimicry (Paz et al., 2022). This process facilitates the rapid transmission of emotions through sensory channels, creating emotional synchrony. Furthermore, perceived attractiveness enhances emotional connection or a sense of presence, prompting instinctive emotional projections. For example, the warm voice and gentle movements of a virtual companion robot may directly alleviate a user’s loneliness or trigger feelings of joy, bypassing rational analysis (Odekerken-Schröder et al., 2020). This emotional resonance is driven by physiological feedback and emotional immersion, with attractiveness playing a central role in fostering the user’s direct sharing of the virtual influencers emotions. Thus, we propose the following hypothesis:

*H5*: Perceived attractiveness is positively correlated with affective empathy.

#### 3.2.2 Perceived authenticity and affective empathy

The authenticity of emotional expression directly activates affective empathy through physiological synchrony and emotional immersion (Salminen et al., 2019). When the emotional displays of virtual influencers align with human instinctive recognition of authenticity, they trigger the user’s mirror neuron system and autonomic physiological responses, creating a feedback loop of emotional contagion. This perception of authenticity also fosters emotional presence, breaking down the human-machine barrier and prompting users to view the virtual influencers as an emotional entity rather than simply a tool (Koles et al., 2024; Lee et al., 2025). This shift encourages the formation of instinctive emotional connections, such as empathetic care or shared joy. The core of affective empathy lies in the embodied resonance triggered by authenticity, where emotional transmission bypasses cognitive processing and directly reaches the user’s affective level (Brinck, 2018). Based on this, we propose the following hypothesis:

*H6*: Perceived authenticity is positively correlated with affective empathy.

#### 3.2.3 Perceived escapism and affective empathy

Through interactions with virtual influencers, users experience a unique sense of escapism, often accompanied by a deep understanding and emotional resonance with the virtual entity’s emotional expressions (Park S. et al., 2021). This emotional connection is not only grounded in shared experiences or emotional states but also heavily relies on the user’s identification with and emotional resonance to the virtual influencers expressive content. When users find resonance in the virtual influencers content, they are more likely to form an emotional bond, leading to a deeper empathic experience. Furthermore, this sense of escapism may encourage users to utilize the emotional expressions of virtual influencers as a tool for emotional regulation. In this process, empathy not only helps users achieve emotional balance but also provides psychological comfort, enabling them to better manage inner anxiety and stress (Ekman and Halpern, 2015; Han et al., 2022). The emotional support and psychological comfort derived from these interactions are significant factors in why users continue to engage with virtual influencers (Jin, 2023). Therefore, this study posits that escapism plays a crucial role in fostering emotional connection and empathy between users and virtual influencers. Based on this, we propose the following hypothesis:

*H7*: Perceived escapism is positively correlated with affective empathy.

#### 3.2.4 Perceived presence and affective empathy

When users feel that their existence is recognized and valued by a community, they are more likely to resonate with the emotional expressions of virtual influencers. This resonance is not solely based on shared emotions but also reflects a deeper emotional connection, as the recognition strengthens the emotional bond between users and virtual influencers. Additionally, the sense of presence fosters users’ interaction and engagement within virtual communities (Cummings et al., 2022; Wellman and Gulia, 1999). This increased social interaction provides more opportunities and contexts for affective empathy. As users engage in more frequent and meaningful interactions, they gain a better understanding of and resonance with others’ emotional states. In this process, the sense of presence acts as a motivational mechanism, encouraging users to participate more actively in virtual community activities—whether through communication, sharing experiences, or expressing emotions—thereby continually reinforcing emotional connections with others (Arsenyan and Mirowska, 2021). Based on the above theoretical analysis, this study suggests that the sense of presence plays a crucial role not only as an intrinsic psychological motivation for users’ well-being but also in effectively fostering affective empathy toward virtual influencers. Therefore, we propose the following hypothesis:

*H8*: Perceived presence is positively correlated with affective empathy.

### 3.3 Cognitive empathy, affective empathy and user engagement

Previous studies have established the significant role of high levels of cognitive empathy in promoting user engagement (Shin, 2018; Ventura et al., 2020). Cognitive empathy enables users to gain a deeper understanding of the emotional states and intentions of content creators, fostering a stronger emotional connection with the content. This connection, in turn, drives positive behavioral responses, such as purchase intentions (Malär et al., 2011). The strength of cognitive empathy lies in its ability to help users transcend virtual boundaries and form a mind-to-mind connection with content creators (Ioannidou and Konstantikaki, 2008). When users can accurately comprehend and resonate with the emotions and intentions conveyed by virtual influencers, they not only develop greater trust in the virtual entity but are also more willing to engage with virtual communities, offering insights and feedback. This trust is central to user engagement, as it lowers psychological barriers, making users more open to accepting and interacting with virtual content (Pera and Viglia, 2016). Similarly, affective empathy enhances users’ emotional investment and satisfaction, significantly promoting their participation in virtual communities (Jung and Im, 2021). Affective empathy strengthens the emotional bond between users and virtual influencers, leading to increased willingness to interact and share personal insights (Paiva et al., 2017). These interactions extend beyond mere information exchange and develop into profound emotional engagement, reinforcing users’ ongoing participation in virtual communities. Based on the above theoretical analysis, we propose the following hypotheses:

*H9*: Cognitive empathy is positively related to user engagement.

*H10*: Affective empathy is positively related to user engagement.

## 4 Research methods

### 4.1 Participants

We recruited online participants through the Credamo,^1^ a reputable professional data collection platform in China (Tang and Ning, 2023). The data collection market on Credamo offers precise positioning services to ensure survey relevance and accuracy. Therefore, we implemented stringent screening criteria, including selecting participants with a historical response acceptance rate of over 80% and experienced in utilizing Sina Weibo. Additionally, we required participants to allocate at least 30 min for completing the entire survey questionnaire to guarantee data quality and participant commitment. Throughout the one-month data collection period, we received a total of 125 questionnaires for response. After conducting preliminary screening of the questionnaires, we excluded invalid samples that failed attention testing questions (e.g., participants who did not select “strongly agree” as their answer) and those with excessively long completion times (over 180 min). Ultimately, we obtained 118 valid participants.

The basic information of the participants is shown in Table 1. Among all valid survey samples, 38.14% were males and 61.86% were females, with a concentration of ages between 21 and 25 years old (accounting for 50%). At the same time, the majority of respondents (86.44%) had known the virtual influencers before participating in this survey, suggesting that virtual influencers have great potential among young people. In addition, the majority of participants are relatively active users who participate in Weibo interactions. Among them, the frequency of liking, commenting, and sharing others’ Weibo accounts for the highest average of 2 to 7 times per week, accounting for 42.37%.

**Table 1:** The basic information of the participants.

Demographic	Category	Count	Percentage
Gender	Male	45	38.14%
	Female	73	61.86%
Age	Below 20	30	25.42%
	21–25	59	50.00%
	26–30	13	11.02%
	31–35	16	13.56%
	Above 35	0	0.00%
Education	High school or below	6	5.08%
	Bachelor’s degree	71	60.17%
	Master’s degree	29	24.58%
	Doctor’s degree	12	10.17%
Ever know of virtual influencers	Yes	102	86.44%
	No	16	13.56%
The frequency of liking, commenting, or forwarding others on Weibo	Once a week or less	12	10.17%
	2 to 7 times per week	50	42.37%
	8–14 times per week	34	28.81%
	15 or more times per week	22	18.64%

### 4.2 Stimuli

In the present study, we embarked on the initial step by acquiring the posts of all virtual influencers active on Sina Weibo, a leading social media platform in China, as recorded up until November 30, 2023. Subsequently, deep learning algorithms were employed to categorize the emotional expression of Weibo posts into six distinct classifications, including happy, angry, sad, fear, surprise and neutral emotion. For the purpose of our experiment, we meticulously selected five Weibo posts from each classified emotion to serve as experimental stimuli. To mitigate potential biases arising from ancillary information, we systematically removed pictures and videos, from the Weibo posts, converting emoticons to their textual equivalents.

### 4.3 Measures

All variables in this study were quantified using a 7-point Likert scale, ranging from 1 (strongly disagree) to 7 (strongly agree), with the specifics of the measurement items for each variable detailed in Appendix A. In particular, the construct of perceived attractiveness, drawing upon the work of Chuah et al. (2020) and Skadberg and Kimmel (2004), was assessed using two items—namely attractiveness and interest. Perceived authenticity was evaluated through four items, based on the research conducted by Kim and Baek (2024) and Li and Ma (2024). Perceived escapism was measured by four items, derived from the studies of Gao et al. (2017) and Wu and Holsapple (2014). Perceived presence was gauged using four items, following the methodologies of Obeidat et al. (2020) and Wang et al. (2019). Additionally, cognitive empathy and affective empathy were each measured with four items, based on the research findings of Cummings et al. (2022) and Shen (2010). Lastly, user engagement intention was explored based on Xu et al. (2020)’s research on users’ intentions to “like,” extended to also encompass intentions to comment and share, comprising a total of nine items. In addition, to ensure clarity and comprehensibility of the content of the experimental stimuli and the scale questions, a preliminary experiment was conducted. This experiment engaged 30 frequent social media users. Detailed analysis of feedback from these pre-experiment participants confirmed the scale’s outstanding performance in terms of reliability and validity. This evidence underscores the robust foundation of our scale for application in the subsequent formal experimental phases.

### 4.4 Procedure

In this study, we adopted a within-subjects design involving 118 participants, who were exposed to six different types of stimuli. The online experiment yielded a total of 708 observations. Prior to the commencement of the experiment, participants were given a succinct introduction to human-likeness virtual influencers and their influence on social media platforms, accompanied by illustrative images of these virtual entities to solidify their understanding of the research context. Following an overview of the experimental protocols and confidentiality guidelines, participants proceeded to engage with the experimental stimuli and subsequently complete a questionnaire designed for this study. To ensure that the emotional expressions embedded in the stimuli were perceived as intended, a manipulation check was embedded within the main questionnaire. After viewing each Weibo post, participants were asked to identify the emotional tone of the post by selecting one of six options: happiness, anger, sadness, fear, surprise, or neutrality. This task was presented as part of the survey flow to avoid drawing attention to the manipulation. The results of the manipulation check revealed a high level of consistency between participants’ responses and the intended emotional categories derived from the deep learning classification. Across all stimuli, the average classification accuracy was 90.32%, and the overall agreement was statistically significant, indicating that participants correctly perceived the emotional expression conveyed by the stimuli. To mitigate potential sequence and fatigue effects, the presentation of stimuli corresponding to each emotional category was randomized across participants’ interfaces. Each advertisement image remained visible for a duration of 1 min. Given the challenges associated with real-time monitoring in an online setting, we introduced attention check questions beneath each stimulus (e.g., “This question is designed to detect your attention; please select ‘strongly agree’”) as a measure to ensure participants’ attentiveness and engagement throughout the experiment. Upon concluding the experimental session, participants were requested to fill out a demographic questionnaire.

## 5 Data analysis and results

In order to comprehensively understand the nuanced and intricate dynamics within these relationships, the research commences with an examination of the linear impacts of perceived attractiveness, authenticity, escapism, and presence on cognitive empathy using linear regression analysis. Following this, the study delves into the aggregated influence of these user perception interactions on cognitive empathy. Furthermore, the variance in the effects of these user perceptions on cognitive empathy across different emotional expression is scrutinized through subgroup regression analysis. This methodological framework is similarly applied to assess the relationship between user perception and affective empathy. Finally, we expanded our exploration scope to understand how cognitive empathy and affective empathy affect user engagement, thereby providing a comprehensive analysis of the pathways through which these perception dimensions interact with user participation.

### 5.1 Descriptive statistics

Table 2 presents the descriptive statistics for the key variables examined in this study. Participants reported moderate to high levels of perceived attractiveness, authenticity, and both cognitive and affective empathy, with mean scores ranging from 4.732 to 5.028 on a 7-point scale. Perceived escapism and presence were rated somewhat lower, with mean scores of 3.957 and 4.244, respectively. User engagement was reported at moderate levels (*M* = 4.127), with the highest variability observed among all variables (*SD* = 1.760). Overall, the results indicate a diverse range of participant experiences, with affective empathy emerging as the most prominently reported dimension.

**Table 2:** Descriptive statistics of variables.

Variables	Mean	SD	Min	Max
Perceived attractiveness	4.825	1.630	1.000	7.000
Perceived authenticity	4.842	1.446	1.000	7.000
Perceived escapism	3.957	1.657	1.000	7.000
Perceived presence	4.244	1.577	1.000	7.000
Cognitive empathy	4.732	1.471	1.000	7.000
Affective empathy	5.028	1.397	1.000	7.000
User engagement	4.127	1.760	1.000	7.000

### 5.2 The relationship between user perception and cognitive empathy

#### 5.2.1 Linear effects

Firstly, we conducted reliability and validity assessments on the scale, with the findings presented in Appendix A. It was observed that the Cronbach’s *α* for all variables surpassed 0.9, Composite Reliability (CR) values exceeded 0.7, and factor loading coefficients for all variables were above 0.5. Additionally, Average Variance Extracted (AVE) values exceeded 0.4, and CR values were above 0.6. Concurrently, Appendix B revealed that the Heterotrait-Monotrait (HTMT) ratio for all variable combinations remained below 0.9. These results suggest robust internal consistency, high reliability, adequate convergent validity, and solid discriminant validity of the scale utilized in this investigation (Fornell and Larcker, 1981; Hair, 2009; Henseler et al., 2015; Kline, 2014; Voorhees et al., 2016).

Subsequently, utilizing a within-subject design, we developed the mixed linear regression models employing Stata 17. Prior to conducting mixed regression analyses, multicollinearity tests were performed on all independent variables to mitigate the influence of high correlations among these variables on the model’s outcomes, in accordance with the methodology proposed by Kroll and Song (2013). The variance inflation factor (VIF) for all independent variables remained below 10, and the tolerance levels exceeded 0.1, demonstrating the absence of significant multicollinearity issues within our model (Daoud, 2017).

Following the establishment of the mixed linear regression model, the findings presented in column (1) of Table 3 revealed significant outcomes. It was observed that perceived attractiveness markedly enhanced cognitive empathy (*β* = 0.069, *p* < 0.05). Similarly, perceived authenticity was found to exert a significantly positive effect on cognitive empathy (*β* = 0.450, *p* < 0.01). Perceived presence also displayed a significant positive correlation with cognitive empathy (*β* = 0.450, *p* < 0.01). However, no substantial association between perceived escapism and cognitive empathy was detected. Consequently, these results underscore the critical roles of perceived attractiveness, authenticity, and presence in facilitating cognitive empathy among users, supporting H1, H2 and H4.

**Table 3:** The mixed regressions between user perception and cognitive empathy.

Variables	(1)	(2)
Perceived attractiveness	0.069**	−0.118
	(2.413)	(−1.180)
Perceived authenticity	0.450***	0.549***
	(13.705)	(6.555)
Perceived escapism	0.010	0.007
	(0.315)	(0.059)
Perceived presence	0.321***	0.492***
	(8.549)	(3.333)
Perceived attractiveness × Perceived authenticity		−0.005
		(−0.259)
Perceived attractiveness × Perceived escapism		0.061***
		(3.141)
Perceived attractiveness × Perceived presence		0.002
		(0.099)
Perceived authenticity × Perceived escapism		−0.021
		(−0.821)
Perceived authenticity × Perceived presence		−0.001
		(−0.042)
Perceived escapism × Perceived presence		−0.045**
		(−2.099)
Constant	0.558***	0.380
Control variables	Yes	Yes
Fixed effects	Yes	Yes

***p < 0.01, **p < 0.05, *p < 0.1.

#### 5.2.2 Interaction effects

The exploration of interaction effects among perceived attractiveness, authenticity, escapism, and presence on cognitive empathy yielded insightful findings, as depicted in column (2) of Table 3. Notably, the interaction between perceived attractiveness and perceived escapism positively influenced cognitive empathy (*β* = 0.061, *p* < 0.01), suggesting that the synergistic effect of finding an aspect attractive while engaging in escapism could amplify cognitive empathy. In contrast, the interaction between perceived escapism and perceived presence negatively affected cognitive empathy (*β* = −0.045, *p* < 0.01), indicating a potential diminution in cognitive empathy when individuals pursue escapism amidst a pronounced sense of presence.

#### 5.2.3 Subgroup analysis

Further, subgroup analysis was conducted to explore the path differences in the relationship between user perception and cognitive empathy across various emotional expressions, as detailed in Table 4. Specifically, perceived attractiveness significantly enhanced cognitive empathy when the virtual influencers’ posts conveyed happy (*β* = 0.169, *p* < 0.05) or fear (*β* = 0.219, *p* < 0.05) emotions. Notably, when compared to the effects under angry (*diff* = 0.210, *p* < 0.1) or sad (*diff* = 0.283, *p* < 0.05) emotions, perceived attractiveness in the context of fear elicited higher levels of cognitive empathy. Moreover, perceived authenticity consistently exerted a significant positive influence on cognitive empathy, irrespective of the emotional state of the post. However, interestingly, these differences did not reach statistical significance across different sentiment types. Furthermore, a negative association between perceived escapism and cognitive empathy was only observed in the context of angry emotion (*β* = −0.151, *p* < 0.1), suggesting that the sense of escapism associated with angry emotion reduced cognitive empathy. Additionally, perceived presence significantly impacted cognitive empathy positively, regardless of the emotional state. It was particularly noted that perceived presence in the contexts of sad (*diff* = −0.222, *p* < 0.1) and fear (*diff* = −0.222, *p* < 0.1) emotions triggered lower levels of cognitive empathy compared to a neutral state.

**Table 4:** Subgroup analysis on path differences in the links of user perception and cognitive empathy.

Path	Item 1	Item 2	Coef 1.	Coef 2.	Difference (1–2)	Chi^2^	*p*-value
Perceived attractiveness → Cognitive empathy	Neutral	Happy	0.026	0.169**	−0.143	0.60	0.437
	Neutral	Angry	0.026	0.009	0.017	0.00	0.953
	Neutral	Sad	0.026	−0.064	0.090	0.29	0.590
	Neutral	Fear	0.026	0.219**	−0.193	1.69	0.193
	Neutral	Surprise	0.026	0.043	−0.017	0.00	0.978
	Happy	Angry	0.169**	0.009	0.160	0.95	0.329
	Happy	Sad	0.169**	−0.064	0.233	2.40	0.122
	Happy	Fear	0.169**	0.219**	−0.050	0.23	0.633
	Happy	Surprise	0.169**	0.043	0.126	0.76	0.385
	Angry	Sad	0.009	−0.064	0.073	0.34	0.558
	Angry	Fear	0.009	0.219**	−0.210	2.81	0.094*
	Angry	Surprise	0.009	0.043	−0.034	0.01	0.918
	Sad	Fear	−0.064	0.219**	−0.283	5.85	0.016**
	Sad	Surprise	−0.064	0.043	−0.107	0.47	0.494
	Fear	Surprise	0.219**	0.043	0.176	2.36	0.124
Perceived authenticity → Cognitive empathy	Neutral	Happy	0.481***	0.547***	−0.066	0.26	0.613
	Neutral	Angry	0.481***	0.630***	−0.149	0.66	0.418
	Neutral	Sad	0.481***	0.630***	−0.149	0.78	0.378
	Neutral	Fear	0.481***	0.381***	0.100	0.38	0.538
	Neutral	Surprise	0.481***	0.516***	−0.035	0.01	0.941
	Happy	Angry	0.547***	0.630***	−0.083	0.03	0.858
	Happy	Sad	0.547***	0.630***	−0.083	0.07	0.791
	Happy	Fear	0.547***	0.381***	0.166	1.16	0.282
	Happy	Surprise	0.547***	0.516***	0.031	0.16	0.685
	Angry	Sad	0.630***	0.630***	0.000	0.01	0.905
	Angry	Fear	0.630***	0.381***	0.249	2.17	0.141
	Angry	Surprise	0.630***	0.516***	0.114	0.43	0.511
	Sad	Fear	0.630***	0.381***	0.249	2.32	0.128
	Sad	Surprise	0.630***	0.516***	0.114	0.53	0.466
	Fear	Surprise	0.381***	0.516***	−0.135	0.42	0.516
Perceived escapism → Cognitive empathy	Neutral	Happy	−0.049	0.029	−0.078	0.18	0.674
	Neutral	Angry	−0.049	−0.151*	0.102	0.39	0.533
	Neutral	Sad	−0.049	0.083	−0.132	1.00	0.317
	Neutral	Fear	−0.049	0.041	−0.090	0.43	0.514
	Neutral	Surprise	−0.049	0.007	−0.056	0.22	0.642
	Happy	Angry	0.029	−0.151*	0.180	1.21	0.272
	Happy	Sad	0.029	0.083	−0.054	0.32	0.573
	Happy	Fear	0.029	0.041	−0.012	0.04	0.839
	Happy	Surprise	0.029	0.007	0.022	0.00	0.966
	Angry	Sad	−0.151*	0.083	−0.234	3.29	0.070*
	Angry	Fear	−0.151*	0.041	−0.192	2.03	0.154
	Angry	Surprise	−0.151*	0.007	−0.158	1.33	0.249
	Sad	Fear	0.083	0.041	0.042	0.16	0.689
	Sad	Surprise	0.083	0.007	0.076	0.27	0.602
	Fear	Surprise	0.041	0.007	0.034	0.03	0.874
Perceived presence → Cognitive empathy	Neutral	Happy	0.453***	0.302**	0.151	1.88	0.170
	Neutral	Angry	0.453***	0.406***	0.047	0.15	0.698
	Neutral	Sad	0.453***	0.231**	0.222	3.03	0.082*
	Neutral	Fear	0.453***	0.231**	0.222	3.17	0.075*
	Neutral	Surprise	0.453***	0.261**	0.192	1.38	0.239
	Happy	Angry	0.302**	0.406***	−0.104	0.64	0.422
	Happy	Sad	0.302**	0.231**	0.071	0.09	0.770
	Happy	Fear	0.302**	0.231**	0.071	0.16	0.690
	Happy	Surprise	0.302**	0.261**	0.041	0.00	0.992
	Angry	Sad	0.406***	0.231**	0.175	1.20	0.274
	Angry	Fear	0.406***	0.231**	0.175	1.36	0.244
	Angry	Surprise	0.406***	0.261**	0.145	0.51	0.477
	Sad	Fear	0.231**	0.231**	0.000	0.01	0.903
	Sad	Surprise	0.231**	0.261**	−0.030	0.07	0.788
	Fear	Surprise	0.231**	0.261**	−0.030	0.13	0.713

***p < 0.01, **p < 0.05, *p < 0.1.

### 5.3 The relationship between user perception and affective empathy

#### 5.3.1 Linear effects

The mixed regression analyses exploring the relationship between user perceptions and affective empathy are reported in Table 5, Column (1). The analyses revealed that perceived authenticity significantly positively influenced affective empathy (*β* = 0.412, *p* < 0.01). In a similar vein, perceived presence was found to significantly enhance affective empathy (*β* = 0.305, *p* < 0.01). Conversely, no significant associations were observed between perceived attractiveness and affective empathy, nor between perceived escapism and affective empathy. These findings underscore the importance of perceived authenticity and presence as key determinants of affective empathy, supporting H6 and H8.

**Table 5:** The mixed regressions between user perception and affective empathy.

Variables	(1)	(2)
Perceived attractiveness	0.048	−0.187*
	(1.601)	(−1.856)
Perceived authenticity	0.412***	0.764***
	(12.018)	(8.921)
Perceived escapism	0.010	−0.115
	(0.290)	(−0.963)
Perceived presence	0.305***	0.530***
	(7.798)	(3.518)
Perceived attractiveness * Perceived authenticity		−0.021
		(−1.034)
Perceived attractiveness * Perceived escapism		0.092***
		(4.730)
Perceived attractiveness * Perceived presence		0.007
		(0.269)
Perceived authenticity * Perceived escapism		−0.047*
		(−1.766)
Perceived authenticity * Perceived presence		−0.030
		(−1.168)
Perceived escapism * Perceived presence		−0.025
		(−1.123)
Constant	1.280***	0.778**
	(6.318)	(2.438)
Control variables	Yes	Yes
Fixed effects	Yes	Yes

***p < 0.01, **p < 0.05, *p < 0.1.

#### 5.3.2 Interaction effects

Further investigations into the interaction effects among perceived attractiveness, authenticity, escapism, and presence on affective empathy were presented in Column (2) of Table 5. Notably, the interaction between perceived attractiveness and escapism was shown to have a significant positive effect on affective empathy (*β* = 0.092, *p* < 0.01). This finding suggests that the influences of perceived attractiveness and escapism on affective empathy are not merely additive but synergistic, enhancing affective empathy when combined. Conversely, the interaction between perceived authenticity and escapism negatively impacted affective empathy (*β* = −0.047, *p* < 0.1), indicating that a simultaneous high perception of authenticity and escapism could lead to a reduction in affective empathy. These interaction effects highlight the nuanced and contingent nature of how perceived attractiveness and escapism influence affective empathy, depending on their interplay with other perceived qualities.

#### 5.3.3 Subgroup analysis

The analysis of path differences in the relationship between user perception and affective empathy across various emotional expressions revealed notable findings, as presented in Table 6. It was observed that perceived attractiveness significantly bolstered affective empathy when the virtual influencers’ posts depicted fear emotion (*β* = 0.262, *p* < 0.05). Moreover, this effect under fear emotion was significantly greater than under happiness (*diff* = 0.321, *p* < 0.05), angry (*diff* = 0.219, *p* < 0.1), sad (*diff* = 0.293, *p* < 0.05) and surprise (*diff* = 0.371, *p* < 0.05) emotions. Notably, perceived authenticity had a consistent and significant positive impact on affective empathy, regardless of the posts’ emotional state. Specifically, perceived authenticity associated with happiness (diff = 0.319, *p* < 0.1) and sad (*diff* = 0.271, *p* < 0.1) elicited higher levels of affective empathy compared to a fear state. Furthermore, perceived escapism significantly diminished affective empathy in the context of posts conveying angry emotion (*β* = −0.234, *p* < 0.05), with a more pronounced detriment compared to fear emotion (*diff* = −0.276, *p* < 0.1). Additionally, perceived presence positively influenced affective empathy across all emotional states. Particularly, its effect was significantly more pronounced under surprise emotion compared to a neutral state (*diff* = 0.277, *p* < 0.1).

**Table 6:** Subgroup analysis on path differences in the links of user perception and affective empathy.

Path	Item 1	Item 2	Coef 1.	Coef 2.	Difference (1–2)	Chi^2^	*P*-value
Perceived attractiveness → Affective empathy	Neutral	Happy	0.052	−0.059	0.111	0.30	0.581
	Neutral	Angry	0.052	0.043	0.009	0.00	0.959
	Neutral	Sad	0.052	−0.031	0.083	0.22	0.640
	Neutral	Fear	0.052	0.262**	−0.210	1.36	0.243
	Neutral	Surprise	0.052	−0.109	0.161	0.76	0.385
	Happy	Angry	−0.059	0.043	−0.102	0.49	0.482
	Happy	Sad	−0.059	−0.031	−0.028	0.04	0.842
	Happy	Fear	−0.059	0.262**	−0.321	5.02	0.025**
	Happy	Surprise	−0.059	−0.109	0.050	0.11	0.738
	Angry	Sad	0.043	−0.031	0.074	0.45	0.501
	Angry	Fear	0.043	0.262**	−0.219	3.77	0.052*
	Angry	Surprise	0.043	−0.109	0.152	1.54	0.215
	Sad	Fear	−0.031	0.262**	−0.293	7.40	0.007**
	Sad	Surprise	−0.031	−0.109	0.078	0.45	0.504
	Fear	Surprise	0.262**	−0.109	0.371	9.42	0.002**
Perceived authenticity → Affective empathy	Neutral	Happy	0.505***	0.664**	−0.159	0.76	0.384
	Neutral	Angry	0.505***	0.542***	−0.037	0.07	0.798
	Neutral	Sad	0.505***	0.582***	−0.077	0.30	0.586
	Neutral	Fear	0.505***	0.345***	0.160	1.00	0.317
	Neutral	Surprise	0.505***	0.508***	−0.003	0.00	0.986
	Happy	Angry	0.664**	0.542***	0.122	0.54	0.463
	Happy	Sad	0.664**	0.582***	0.082	0.26	0.613
	Happy	Fear	0.664**	0.345***	0.319	3.12	0.078*
	Happy	Surprise	0.664**	0.508***	0.156	0.78	0.376
	Angry	Sad	0.542***	0.582***	−0.040	0.11	0.738
	Angry	Fear	0.542***	0.345***	0.197	1.93	0.164
	Angry	Surprise	0.542***	0.508***	0.034	0.06	0.803
	Sad	Fear	0.582***	0.345***	0.237	2.94	0.086*
	Sad	Surprise	0.582***	0.508***	0.074	0.31	0.578
	Fear	Surprise	0.345***	0.508***	−0.163	1.13	0.288
Perceived escapism → Affective empathy	Neutral	Happy	−0.118	−0.074	−0.044	0.07	0.796
	Neutral	Angry	−0.118	−0.234**	0.116	0.59	0.444
	Neutral	Sad	−0.118	−0.095	−0.023	0.02	0.876
	Neutral	Fear	−0.118	0.042	−0.160	0.93	0.335
	Neutral	Surprise	−0.118	−0.048	−0.070	0.20	0.656
	Happy	Angry	−0.074	−0.234**	0.160	1.44	0.230
	Happy	Sad	−0.074	−0.095	0.021	0.03	0.868
	Happy	Fear	−0.074	0.042	−0.116	0.60	0.438
	Happy	Surprise	−0.074	−0.048	−0.026	0.03	0.854
	Angry	Sad	−0.234**	−0.095	−0.139	1.85	0.174
	Angry	Fear	−0.234**	0.042	−0.276	4.67	0.031**
	Angry	Surprise	−0.234**	−0.048	−0.186	2.58	0.108
	Sad	Fear	−0.095	0.042	−0.137	1.27	0.261
	Sad	Surprise	−0.095	−0.048	−0.047	0.18	0.668
	Fear	Surprise	0.042	−0.048	0.090	0.46	0.499
Perceived presence → Affective empathy	Neutral	Happy	0.205**	0.304**	−0.099	0.35	0.557
	Neutral	Angry	0.205**	0.425***	−0.220	1.67	0.196
	Neutral	Sad	0.205**	0.312**	−0.107	0.51	0.474
	Neutral	Fear	0.205**	0.257**	−0.052	0.10	0.757
	Neutral	Surprise	0.205**	0.482***	−0.277	2.73	0.098*
	Happy	Angry	0.304**	0.425***	−0.121	0.45	0.503
	Happy	Sad	0.304**	0.312**	−0.008	0.00	0.962
	Happy	Fear	0.304**	0.257**	0.047	0.07	0.792
	Happy	Surprise	0.304**	0.482***	−0.178	1.00	0.318
	Angry	Sad	0.425***	0.312**	0.113	0.49	0.485
	Angry	Fear	0.425***	0.257**	0.168	0.87	0.351
	Angry	Surprise	0.425***	0.482***	−0.057	0.10	0.748
	Sad	Fear	0.312**	0.257**	0.055	0.12	0.733
	Sad	Surprise	0.312**	0.482***	−0.170	1.14	0.286
	Fear	Surprise	0.257**	0.482***	−0.225	1.61	0.205

***p < 0.01, **p < 0.05, *p < 0.1.

### 5.4 The relationship between cognitive empathy, affective empathy and user engagement

A mixed model analysis was conducted using Stata 17 to examine the relationship between cognitive empathy, affective empathy, and user engagement. The findings indicated that both cognitive empathy (*β* = 0.510, *p* < 0.001) and affective empathy (*β* = 0.272, *p* < 0.001) significantly enhanced user engagement (seen on Table 7), supporting H9 and H10. Moreover, the results showed that there was no significant association between cognitive empathy and affective empathy.

**Table 7:** The mixed regressions between cognitive empathy, affective empathy, and user engagement.

Variables	(1)	(2)
Cognitive empathy	0.510***	0.407***
	(13.038)	(4.242)
Affective empathy	0.272***	0.197**
	(6.793)	(2.637)
Cognitive empathy * Affective empathy		0.020
		(1.192)
Constant	0.579**	0.921**
	(1.989)	(2.241)
Control variables	Yes	Yes
Fixed effects	Yes	Yes

***p < 0.01, **p < 0.05, *p < 0.1.

Further, the mediation effects of cognitive and affective empathy were investigated employing a bootstrap approach (with 5,000 bootstrapping samples). The outcomes were presented in Table 8. Specifically, the indirect effect of perceived attractiveness on user engagement through cognitive empathy was measured at 0.282, with a 95% confidence interval ranging from 0.224 to 0.339. The indirect effect of perceived authenticity on user engagement via cognitive empathy was 0.499, with a 95% confidence interval from 0.401 to 0.598. The indirect effect of perceived escapism on user engagement through cognitive empathy was 0.278, with a confidence interval of 0.227 to 0.330. Furthermore, the indirect effect of perceived presence on user engagement via cognitive empathy was found to be 0.241, with a 95% confidence interval ranging from 0.172 to 0.310, indicating that cognitive empathy mediates the relationship between user perception (i.e., perceived attractiveness, perceived authenticity, perceived escapism, and perceived presence) and user engagement. Additionally, the indirect effect of perceived attractiveness on user engagement through affective empathy was 0.178, with a confidence interval of 0.130 to 0.226. The indirect effect of perceived authenticity on user engagement via affective empathy was 0.264, with a 95% confidence interval of 0.167 to 0.362. The indirect effect of perceived escapism on user engagement through affective empathy was 0.183, with a confidence interval of 0.140 to 0.226. Lastly, the indirect effect of perceived presence on user engagement via affective empathy was 0.120, with a 95% confidence interval of 0.063 to 0.177, suggesting that affective empathy also serves as a mediating factor between user perception (i.e., perceived attractiveness, perceived authenticity, perceived escapism, and perceived presence) and user engagement.

**Table 8:** Mediation effect test results.

Path	Indirect effect value	SE	Boot LLCI	Boot ULCI
Perceived attractiveness → cognitive empathy → user engagement	0.282	0.029	0.224	0.339
Perceived authenticity → cognitive empathy → user engagement	0.499	0.050	0.401	0.598
Perceived escapism → cognitive empathy → user engagement	0.278	0.026	0.227	0.330
Perceived presence → cognitive empathy → user engagement	0.241	0.035	0.172	0.310
Perceived attractiveness → affective empathy → user engagement	0.178	0.025	0.130	0.226
Perceived authenticity → affective empathy → user engagement	0.264	0.050	0.167	0.362
Perceived escapism → affective empathy → user engagement	0.183	0.022	0.140	0.226
Perceived presence → affective empathy → user engagement	0.120	0.029	0.063	0.177

## 6 Discussion

This study endeavors to shed light on the antecedents that influence the emotional expression of virtual influencers and their subsequent effects on user empathy and engagement on social media platforms. By empathy model, we delve into the complex interplay between perceived attractiveness, authenticity, escapism, presence, and both cognitive and affective empathy, utilizing a diverse array of analytical methodologies, including linear effects, interaction effects, and subgroup analysis.

Initially, our examination of linear effects reveals that perceived authenticity and presence significantly enhance both cognitive and affective empathy, aligning with existing scholarly discourse (Li and Ma, 2024; Quach et al., 2024). This underscores the notion that authenticity, as a critical aspect of engaging with virtual influencers, may prompt users to replicate and assimilate processes that involve comprehending and empathizing with the emotional expressions of virtual influencers. Users might relate these emotional expressions to their personal experiences, thereby intensifying their empathy toward the emotions conveyed by virtual influencers. Presence, or the user’s perceived immersion within a virtual environment, similarly enhances empathy by allowing users to more fully place themselves within the context of virtual influencers, thereby enriching the emotional exchange. Moreover, our findings indicate that perceived attractiveness significantly contributes to the enhancement of cognitive empathy. This implies that within the realm of virtual influencers, perceived attractiveness extends beyond mere appearance to encompass the allure of character behavior, language, and emotional articulation (Roccapriore and Pollock, 2023; Shen et al., 2022). When users discern a high level of attractiveness in virtual influencers, they are more inclined to devote time and effort to understanding and empathizing with their emotional expressions, thus elevating cognitive empathy levels. Notably, our research demonstrates that perceived escapism does not influence cognitive and affective empathy. This deviation suggests that the sense of escape, often conceptualized as a psychological state wherein an individual seeks to detach from daily life pressures through certain behaviors, may be more closely associated with the desire to evade negative emotions or stressful situations rather than directly involving the cognition or perception of others’ emotional states. Consequently, perceived escapism does not appear to directly bolster an individual’s capacity for cognitive empathy as well as affective empathy.

In terms of the interaction effects, our findings reveal that perceived attractiveness and escapism, when considered together, can enhance both cognitive and affective empathy among users. Notably, when the interaction term is included, the main effect of perceived attractiveness on affective empathy shifts from significantly positive to significantly negative, indicating a suppression or reversal effect in the presence of escapism. Concurrently, the interaction between attractiveness and escapism exerts a significant positive impact on affective empathy. This indicates that the effect of attractiveness on affective empathy is not linear but contingent upon the level of escapism. It suggests that users are more adept at understanding and empathizing with the thoughts and emotions of others when they experience both the allure of certain aspects and the desire for escapism. This points to a compelling synergistic effect, where factors of attraction within escapism experiences can amplify an individual’s cognitive empathy, likely because this amalgamation offers a more immersive and engaging experience that fosters an enhanced comprehension and resonance with others’ emotional states (Fu et al., 2023; Guan et al., 2024). This highlights that under specific conditions, namely the presence of attractiveness, escapism emerges as a significant enhancer of affective empathy. Furthermore, our analysis of the interaction between perceived presence and escapism reveals a significant negative impact on cognitive empathy. This suggests that while the sense of presence independently acts as a robust promoter of cognitive empathy, its combination with escapism could diminish an individual’s cognitive empathic capabilities. This finding unveils a critical conditional effect, whereby the positive influence of presence on cognitive empathy may be mitigated by escapism. This could occur because individuals experiencing high levels of presence alongside a desire to escape face an internal dissonance that may divert their focus and lessen their capacity for cognitive processing of others’ emotional states (Allen et al., 2022; Kahn, 1990). Additionally, our analysis suggests that a heightened confluence of perceived authenticity and escapism may lead to a reduction in affective empathy. This reduction could stem from a psychological dissonance between highly authentic experiences and the wish to escape reality, potentially curtailing the development of affective empathy.

Moreover, in the conducted subgroup analysis, the data reveal that perceived attractiveness markedly amplifies both cognitive and affective empathy, especially in scenarios where virtual influencers’ posts are imbued with sentiments of happiness or fear. These observations intimate that attractiveness may act as a catalyst for fostering a deeper emotional connection and comprehension, particularly in situations that provoke robust emotional reactions. Concurrently, our study corroborates existing literature by demonstrating the unvarying positive influence of perceived authenticity on both cognitive and affective empathy, irrespective of the emotional tone of the post. This pattern underscores the pivotal role of authenticity in augmenting emotional engagement, suggesting that users place a high value on authentic content, thereby enabling a more profound empathetic rapport (Ozdemir et al., 2023; Yu et al., 2024). Besides, the analysis identified a negative correlation between perceived escapism and cognitive empathy within the framework of anger-driven emotions, indicating escapism’s potential to attenuate empathy. This observation might suggest that user engagement with content as a means of escapism, especially when the content carries a negative valence, could impair their capability to comprehend and resonate with others’ feelings. This finding lends additional support to the argument that escapism, although a prevalent motive behind engaging with virtual content, may lead to unforeseen effects on emotional processing and empathy (Decety and Lamm, 2006; Stocks et al., 2009). Furthermore, perceived presence significantly impacts empathy, accentuating the importance of immersion and engagement in eliciting an empathetic response (Shin, 2018). This implies that the greater the sense of presence users experience within a virtual environment, the more likely they are to empathize with the content. Importantly, the influence of perceived presence on empathy is observed to fluctuate across different emotional contexts, with specific emotions (e.g., surprise) significantly boosting affective empathy in comparison to a neutral baseline. Such variability suggests that the level of presence experienced by users can intricately interact with the emotional tenor of the content, thereby modulating levels of empathy.

Finally, our research confirms that both cognitive and affective empathy are significant predictors of user engagement, which is consistent with previous studies (Jin, 2023; Mirowska and Arsenyan, 2023). Meanwhile, our study highlights the mediating role of empathy in the relationship between user perceptions and engagement. This underscores the importance of empathy in enhancing user engagement with virtual influencers, suggesting that perceptions of attractiveness, authenticity, escapism, and presence can significantly influence engagement through the mechanism of empathy. For instance, the perception of attractiveness may stimulate cognitive empathy, prompting users to delve into the motivations and emotions underlying the creation of engaging content. Similarly, a sense of authenticity could bolster affective empathy, as users perceive the content or experience to be more genuine and credible, thereby facilitating an emotional connection. Furthermore, the notions of escapism and presence can deepen user immersion and engagement by amplifying both cognitive and affective empathy, enabling users to achieve a more profound comprehension and engagement with the content or experience, especially when seeking an escape from reality or experiencing a sense of being there.

## 7 Implications and limitations

### 7.1 Theoretical contributions

Our study makes three principal theoretical contributions to the existing body of literature. Firstly, by examining the relationship between the emotional expression of human-like virtual influencers and user engagement, our research expands the comprehension of user engagement dynamics within social media contexts. While previous investigations have probed the connection between influencer emotional expression and user engagement, the domain of virtual influencers, particularly within the Chinese market context, has remained largely unexplored. Our pioneering efforts to scrutinize the impact of virtual influencers’ emotional expression on user engagement address this lacuna, enriching the scholarly understanding of virtual influencers’ roles on social media platforms.

Secondly, this study elucidates the diverse factors that modulate user empathy and their subsequent influence on user engagement levels, thereby enriching the discourse on user empathy in the context of virtual influencers. Existing literature has underscored the significance of empathy in the dynamics between human-like virtual influencers and users but has frequently overlooked the precise elements that shape empathy. By extending current models of empathy and applying them within the realm of virtual influencers, our research offers novel insights into the formation of emotional connections between users and virtual entities, underpinned by empirical evidence.

Lastly, our investigation uncovers the mechanisms that drive users’ intentions to engage on social media platforms, with a particular focus on the critical mediating roles of cognitive empathy and emotional empathy, leveraging a data-driven methodology. These insights not only deepen the scholarly understanding of user behavior on social media but also provide a solid empirical foundation for boosting user engagement, especially offering strategic recommendations for crafting interactions between users and virtual influencers. Collectively, our findings not only theoretically augment the corpus of knowledge on virtual influencers but also shed light on the profound impact of user empathy and perceptions on engagement intentions. These findings lay a solid foundation for further research and the implementation of strategies involving virtual influencers.

### 7.2 Practical implications

This research offers several practical implications. First, for the operational teams of human-like virtual influencers, recognizing the crucial role of authenticity in enhancing users’ cognitive and emotional empathy is imperative (Ham et al., 2024; Park et al., 2023). Enhancing the emotional expression capabilities of virtual influencers to improve cognitive empathy is also a significant area of focus that should not be overlooked. Simultaneously, leveraging the synergistic effects of attraction and escapism to augment both cognitive and emotional empathy is essential (Zhou et al., 2024). Providing an escape experience while maintaining a high level of attractiveness can deepen user engagement. Additionally, understanding how various emotions, such as happiness, fear, and anger, influence empathy allows for tailoring content to maximize empathy and engagement (Li et al., 2024).

Second, for brands, adopting strategies that utilize human-like virtual influencers who embody high levels of authenticity and attractiveness to develop emotionally resonant marketing campaigns can increase engagement and foster brand loyalty. Brands can also establish long-term partnerships with human-like virtual influencers whose authenticity, emotional appeal, and brand values align, leading to more meaningful and impactful collaborations.

Finally, for social media platforms, to further facilitate the integration of human-like virtual influencers and influencer marketing, focusing on enhancing user experience and incorporating features that strengthen presence and authenticity, such as live streaming, interactive stories, and personalized interactions, is critical to increasing user empathy and engagement. Furthermore, optimizing the recommendation algorithm to prioritize posts from human-like virtual influencers that promote empathy, such as those perceived as genuine or that elicit strong emotional responses, can significantly contribute to creating more meaningful and engaging experiences for users, fostering deeper emotional connections, and enhancing overall engagement.

### 7.3 Limitations and future research directions

This study acknowledges several limitations that merit consideration. Firstly, our analysis predominantly utilized Sina Weibo as the social media platform of choice, potentially constraining the extrapolation of our findings. To enhance the generalizability of the research, future studies are encouraged to undertake cross-platform analyses to uncover distinctions in user attitudes and cognitive perceptions across various platforms. Our investigation primarily concentrated on the influence of virtual influencers’ emotional expression in Weibo texts on user engagement. Subsequent research could broaden this inquiry to assess the effects of multimodal presentations by virtual entities on social media engagement. Moreover, this study was primarily concerned with how emotional expression of human-like virtual influencers affect user engagement. Future investigations could expand to include other semantic aspects of posts (e.g., post topic) and linguistic features (e.g., communication styles).

Secondly, the online experimental design adopted in our study, while facilitating an expanded sample reach, introduces potential risks to the integrity of experimental completion, despite incorporating attention checks. Furthermore, the within-subjects experimental setup, though effective in mitigating individual variance, may introduce carryover effects among participants. We recommend future research to consider implementing offline experiments that blend within-subject and between-subject designs to more robustly validate our findings.

Lastly, our experimental framework was tailored to explore the effects of perceived attractiveness, authenticity, escapism, and presence on empathy. Future research endeavors could delve into additional dimensions of individual psychological needs, such as the desire for uniqueness, and their impact on empathy. It is also suggested that subsequent studies explore variations in user perceptions and attitudes toward diverse virtual influencers, to provide a more comprehensive understanding of the dynamics at play.

## 8 Conclusion

This study comprehensively analyzed the factors that affect cognitive empathy and emotional empathy among virtual influencers, as well as their impact on user engagement. The core of our research findings is that perceived authenticity and presence can significantly increase cognitive empathy and affective empathy. Meanwhile, perceived attractiveness increases cognitive empathy, and the impact of perceived attractiveness on affective empathy depends on the degree of perceived escapism. The sub sample analysis further revealed differences in user perception of emotional expression among virtual influencers. The study also emphasizes the crucial mediating role of cognitive empathy and affective empathy in user perception and engagement relationships. With the development of the field of virtual influencers, these insights provide guidance for maintaining the relationship between virtual influencers and users on social media platforms, benefiting practitioners and researchers.

## Data Availability

The raw data supporting the conclusions of this article will be made available by the authors, without undue reservation.

## Footnotes

1
https://www.credamo.com
