# Supplementary material for: How emotional expression in human-like virtual influencers drives user engagement: empathy model and its antecedents
Source: Front Psychol. 2026 Jan 6;16:1544037. doi: 10.3389/fpsyg.2025.1544037 (PMC12816228; doi:10.3389/fpsyg.2025.1544037)
Supplement: Supplementary file 1 [file Supplementary_file_1.docx]

# Appendix

## Appendix A. The reliability and validity assessments on the scale.

| Variables | Item | Factor loading | CR | Cronbach’s $\alpha$ | AVE | References |
| --- | --- | --- | --- | --- | --- | --- |
| Perceived attractiveness  (ATT) | ATT1: I think this content from virtual influencer attractive. | 0.739 | 0.718 | 0.930 | 0.560 | Chuah et al. (2020);  Skadberg and Kimmel (2004) |
|  | ATT2: I think this content from virtual influencer interesting. | 0.758 |  |  |  |  |
| Perceived authenticity  (AUT) | AUT1: I think this virtual influencer behaves the same way as human influencer. | 0.751 | 0.822 | 0.916 | 0.536 | Kim & Baek (2023);  Li and Ma (2023) |
|  | AUT2: I think the content from this virtual influencer is relatable to my daily life. | 0.707 |  |  |  |  |
|  | AUT3: I think this virtual influencer is sincere. | 0.761 |  |  |  |  |
|  | AUT4: I think this virtual influencer is honest. | 0.707 |  |  |  |  |
| Perceived escapism  (ES) | ES1: This content from virtual influencer helps me escape from the world of reality. | 0.811 | 0.881 | 0.932 | 0.649 | Gao et al. (2017);  Wu and Holsapple (2014) |
|  | ES2: This content from virtual influencer helps me escape from problems and pressures. | 0.826 |  |  |  |  |
|  | ES3: This content from virtual influencer helps me escape from things that are unpleasant and worrisome. | 0.820 |  |  |  |  |
|  | ES4: This content from virtual influencer makes me feel as if I am in a different world of reality. | 0.764 |  |  |  |  |
| Perceived presence  (PP) | PP1: This content from virtual influencer provides a sense of belonging. | 0.662 | 0.751 | 0.919 | 0.430 | Obeidat et al. (2020);  Wang et al. (2019) |
|  | PP2: This content from virtual influencer makes it a sense of sociability. | 0.650 |  |  |  |  |
|  | PP3: This content from virtual influencer helps others better understand me. | 0.607 |  |  |  |  |
|  | PP4: This content from virtual influencer would allow others to know me well even if I only met them online. | 0.700 |  |  |  |  |
| Cognitive empathy  (CE) | CE1: I can see the virtual influencer’s point of view. | 0.688 | 0.809 | 0.925 | 0.514 | Cummings et al. (2022);  Shen (2010) |
|  | CE2: I recognize the virtual influencer’s situation. | 0.744 |  |  |  |  |
|  | CE3: I can understand what the virtual influencer was going through in the message. | 0.713 |  |  |  |  |
|  | CE4: The virtual influencer’s reactions to the situation are understandable. | 0.722 |  |  |  |  |
| Affective empathy  (AE) | AE1: The virtual influencer’s emotion is genuine. | 0.819 | 0.865 | 0.929 | 0.617 | Cummings et al. (2022);  Shen (2010) |
|  | AE2: I experienced the same emotion as the virtual influencer when watching this content. | 0.799 |  |  |  |  |
|  | AE3: I was in a similar emotional state as the virtual influencer when watching this content. | 0.738 |  |  |  |  |
|  | AE4: I can feel the virtual influencer’s emotion. | 0.783 |  |  |  |  |
| User engagement  (UE) | UE1: I am willing to click the “like” button about this content. | 0.712 | 0.934 | 0.973 | 0.611 | Xu et al. (2020) |
|  | UE2: The likelihood of me clicking the “like” button in this content is high. | 0.759 |  |  |  |  |
|  | UE3: I will frequently click the “like” button in this content in the future. | 0.786 |  |  |  |  |
|  | UE4: I am willing to review this content. | 0.806 |  |  |  |  |
|  | UE5: The likelihood of me clicking the “comment” button in this content is high. | 0.819 |  |  |  |  |
|  | UE6: I will frequently click the “comment” button in this content in the future. | 0.805 |  |  |  |  |
|  | UE7: I am willing to click the “share” button about this content. | 0.774 |  |  |  |  |
|  | UE8: The likelihood of me clicking the “share” button in this content is high. | 0.785 |  |  |  |  |
|  | UE9: I will frequently click the “share” button in this content in the future. | 0.784 |  |  |  |  |

## Appendix B. The heterotrait-monotrait ratio.

|  | **ATT** | **AUT** | **ES** | **PP** | **CE** | **AE** | **UE** |
| --- | --- | --- | --- | --- | --- | --- | --- |
| **ATT** | **-** |  |  |  |  |  |  |
| **AUT** | 0.763 | **-** |  |  |  |  |  |
| **ES** | 0.741 | 0.650 | **-** |  |  |  |  |
| **PP** | 0.782 | 0.807 | 0.860 | **-** |  |  |  |
| **CE** | 0.719 | 0.878 | 0.673 | 0.839 | **-** |  |  |
| **AE** | 0.659 | 0.840 | 0.593 | 0.769 | 0.887 | **-** |  |
| **UE** | 0.714 | 0.689 | 0.695 | 0.822 | 0.768 | 0.674 | **-** |
